# Cardiovascular Implications of Diabetes Mellitus: A Systematic Literature Review

**DOI:** 10.7759/cureus.109607

**Published:** 2026-05-25

**Authors:** Piyush Kumar Gupta, Anand Sekar G, Ram Diwakar, Niyanta Ashokkumar Panchal, Anshul Kumar, Anupravo Bhaumik

**Affiliations:** 1 Department of Community Medicine, Geetanjali Institute of Medical Sciences, Jaipur, IND; 2 Department of Cardiology, Aarupadai Veedu Medical College, Vinayaka Missions Research Foundation (VMRF-DU), Puducherry, IND; 3 Department of General Medicine, Maharshi Vashishtha Autonomous State Medical College, Basti, Basti, IND; 4 Department of Anesthesia, Dr. N. D. Desai Faculty of Medical Science, Dharmsinh Desai University, Nadiad, IND; 5 Department of Endocrinology and Anesthesiology, Venkateshwar Hospital, New Delhi, IND; 6 Department of General Medicine, Agartala Government Medical College and Govind Ballabh Pant Hospital, Agartala, IND

**Keywords:** cardiovascular disease, diabetes mellitus, glycemic control, heart failure, systematic review

## Abstract

Diabetes mellitus is a chronic metabolic disorder associated with a two- to four-fold higher risk of cardiovascular morbidity and mortality compared with individuals without diabetes due to complex interactions between metabolic, inflammatory, and vascular pathways. Despite extensive research, uncertainties remain regarding the integrated effects of emerging therapies and persistent cardiovascular risk in diabetic populations. This systematic literature review aimed to evaluate the cardiovascular implications of diabetes mellitus by synthesizing evidence on epidemiology, mechanisms, and clinical outcomes. A structured search was conducted across PubMed, Scopus, Web of Science, and Cochrane Library for studies published between 2015 and 2025, using predefined Boolean search terms, including MeSH terms in PubMed, to identify randomized controlled trials and observational studies reporting cardiovascular outcomes; no review protocol was registered prospectively. Eleven studies were included in the final review after searches were conducted for studies published between 2015 and 2025. Data were extracted and synthesized narratively because clinical and methodological heterogeneity, including differences in populations, interventions, outcomes, study designs, and follow-up durations, precluded formal meta-analysis, and I² assessment. The findings indicate that diabetes significantly increases the risk of major adverse cardiovascular events, heart failure, and mortality, driven by hyperglycemia-induced endothelial dysfunction, inflammation, and atherosclerosis. Pharmacological therapies such as SGLT2 inhibitors, GLP-1 receptor agonists, and mineralocorticoid receptor antagonists demonstrated significant reductions in cardiovascular and renal outcomes, while lifestyle interventions contributed to risk reduction. Persistent residual risk highlights incomplete therapeutic control. These findings emphasize the need for integrated and individualized management strategies. This review reinforces the importance of combining pharmacological and lifestyle interventions to effectively reduce cardiovascular complications in diabetes.

## Introduction and background

Diabetes mellitus is a major health concern globally and is associated with substantial morbidity and mortality, largely because it increases the risk of cardiovascular disease, including coronary artery disease, stroke, heart failure, and cardiovascular death [1,2]. The prevention and treatment of cardiovascular disease have improved considerably; however, diabetes remains an independent risk factor for both microvascular and macrovascular complications, contributing to disease progression, adverse clinical outcomes, and increased healthcare burden [3]. Pharmacological interventions such as metformin have demonstrated potential cardioprotective effects beyond glycemic control, although their mechanisms remain multifactorial and incompletely understood in clinical practice [4].

The pathophysiological link between diabetes and cardiovascular disease is mediated through chronic hyperglycemia, insulin resistance, endothelial dysfunction, oxidative stress, inflammation, and dysregulated lipid metabolism [5]. Early clinical observations established that markers such as urinary albumin excretion are strongly associated with cardiovascular risk in diabetic patients, highlighting the role of endothelial damage and vascular permeability as early indicators of systemic vascular dysfunction and disease progression [6]. These mechanisms promote atherosclerosis, plaque instability, and diabetic cardiomyopathy, thereby increasing the likelihood of myocardial infarction, stroke, heart failure, and other adverse cardiovascular outcomes [7,8].

Advancements in molecular research have identified novel pathogenetic mechanisms in type 2 diabetes, including altered insulin signaling and metabolic inflexibility, which play a critical role in accelerating cardiovascular complications and underscore the need for targeted therapeutic strategies [9,10]. Although glucose-lowering, cardioprotective, and lifestyle-based interventions have improved cardiovascular prevention, residual cardiovascular risk remains substantial in patients with diabetes [10,11]. This persistent risk may reflect variability in treatment response, comorbid disease burden, and mechanisms not fully addressed by current therapies. Although prior reviews have addressed diabetes-related cardiovascular epidemiology, specific drug classes, or individual outcomes separately, limited recent evidence has integrated mechanisms, clinical outcomes, therapeutic interventions, residual cardiovascular risk, and treatment-response heterogeneity within a single synthesis. The present review addresses this gap by providing an updated, integrated assessment of cardiovascular implications of diabetes mellitus, with emphasis on therapeutic outcomes and patient-level variability in response.

Objectives of the review

This review aims to provide an integrated synthesis of the cardiovascular implications of diabetes mellitus, primarily focused on therapeutic outcomes while incorporating relevant epidemiological and mechanistic evidence. Specifically, it seeks to identify and characterize key pathophysiological pathways, evaluate pharmacological and lifestyle interventions, summarize major cardiovascular outcomes, and assess heterogeneity in treatment response across patient populations, cardiovascular risk profiles, comorbidities, and intervention classes.

## Review

Methodology

Search Strategy

A systematic literature search was conducted in PubMed, Scopus, Web of Science, and Cochrane Library to identify studies evaluating the association between diabetes mellitus and cardiovascular disease. The search was performed for studies published between January 1, 2015, and December 31, 2025, and was restricted to human studies and English-language articles. The PubMed search strategy included both Medical Subject Headings (MeSH) and free-text terms. The complete PubMed search string was as follows: (“Diabetes Mellitus”[MeSH] OR “diabetes mellitus” OR “type 1 diabetes” OR “type 2 diabetes” OR “T1DM” OR “T2DM”) AND (“Cardiovascular Diseases”[MeSH] OR “cardiovascular disease” OR “cardiovascular complications” OR “coronary artery disease” OR “myocardial infarction” OR “stroke” OR “heart failure” OR “major adverse cardiovascular events” OR “MACE”) AND (“mortality” OR “cardiovascular mortality” OR “hospitalization” OR “clinical outcomes” OR “risk”). The filters applied in PubMed were publication date from January 1, 2015, to December 31, 2025; humans; English language; and adult population, where available.

For Scopus, Web of Science, and Cochrane Library, the search strategy was adapted using title, abstract, and keyword fields. The complete adapted search string was as follows: TITLE-ABS-KEY (“diabetes mellitus” OR “type 1 diabetes” OR “type 2 diabetes” OR “T1DM” OR “T2DM”) AND TITLE-ABS-KEY (“cardiovascular disease” OR “cardiovascular complications” OR “coronary artery disease” OR “myocardial infarction” OR “stroke” OR “heart failure” OR “major adverse cardiovascular events” OR “MACE”) AND TITLE-ABS-KEY (“mortality” OR “cardiovascular mortality” OR “hospitalization” OR “clinical outcomes” OR “risk”). The same publication date, English-language, human-subject, and adult-population filters were applied where available. Boolean operators “AND” and “OR” were used to combine major concepts and synonyms, respectively. Reference lists of potentially eligible articles were also manually screened to identify additional relevant studies. All retrieved references were reviewed for relevance to the cited claims, and sources not directly supporting diabetes-related cardiovascular epidemiology, mechanisms, interventions, or outcomes were replaced or removed. The review was conducted in accordance with the Preferred Reporting Items for Systematic Reviews and Meta-Analyses (PRISMA) 2020 guidelines [12]. No review protocol was registered in PROSPERO or another prospective systematic review registry.

Eligibility Criteria

Inclusion criteria: Studies had to evaluate the association between diabetes mellitus and cardiovascular outcomes with clear clinical relevance. Randomized controlled trials, cohort studies, and case-control studies conducted in adult populations were also eligible. Studies involving type 1 or type 2 diabetes and cardiovascular outcomes, including coronary artery disease, stroke, heart failure, cardiovascular mortality, or major adverse cardiovascular events (MACE), were considered. No minimum sample size or follow-up duration was imposed because eligible studies varied substantially in design and population size; however, studies were required to report extractable cardiovascular outcome data.

Exclusion criteria: Non-comparative studies, editorials, case reports, and studies not written in English were excluded. Studies that lacked clear cardiovascular endpoints or adequate methodology were also excluded. Prior systematic reviews and meta-analyses were excluded from the final study pool because this review aimed to synthesize primary clinical evidence and avoid duplication of already pooled findings. However, relevant systematic reviews and meta-analyses were screened and used for contextual support in the Introduction and Discussion where appropriate. Filtering of records was done before the final selection, and records that did not meet the eligibility criteria were dropped.

Data Extraction and Analysis

To ensure consistency, data were extracted using a standardized data extraction form. Extracted variables included study design, sample size, population characteristics, diabetes type, intervention or exposure, comparator group, follow-up duration, cardiovascular outcomes, and key findings. Data extraction was performed independently by two reviewers, and disagreements were resolved through discussion or consultation with a third reviewer. The primary outcomes of interest were cardiovascular disease incidence, cardiovascular mortality, heart failure hospitalization, and MACE. A quantitative meta-analysis was not performed because the included studies showed substantial clinical, methodological, and outcome heterogeneity, including differences in populations, diabetes type, interventions, comparators, cardiovascular endpoints, study designs, follow-up durations, and reported effect measures. Because the studies did not consistently report comparable effect sizes for a common outcome, formal statistical heterogeneity assessment using I² was not performed. Subgroup analysis or meta-regression was considered but not conducted because the small number of included studies and the lack of comparable pooled estimates would have made such analyses unreliable. Therefore, findings were synthesized narratively and summarized in structured tables to facilitate comparison across studies.

Quality Assessment

The methodological quality of included studies was assessed to determine the reliability of the evidence. Randomized controlled trials were assessed using the Cochrane Risk of Bias 2 tool, and observational studies were assessed using the Newcastle-Ottawa Scale [13,14]. Quality assessment was performed independently by two reviewers, and disagreements were resolved through discussion or consultation with a third reviewer. Evaluation domains included clarity of study objectives, appropriateness of study design, comparability of study groups, accuracy of outcome measurement, control of confounding, and adequacy of follow-up. The overall quality grading was considered during interpretation of the findings, with greater emphasis placed on studies demonstrating stronger methodological rigor. No studies were excluded solely based on quality assessment.

Risk-of-Bias Assessment

Risk of bias was assessed at the study level by examining potential sources of systematic error. Key domains included selection bias, performance bias, detection bias, and reporting bias. The Cochrane Risk of Bias 2 tool was used for randomized controlled trials, while the Newcastle-Ottawa Scale was used for observational studies [13,14]. Particular attention was given to baseline comparability between study groups, selection of study participants, ascertainment of exposure, outcome measurement, adequacy of follow-up, and methods used to control confounding variables. Randomized studies were evaluated based on the adequacy of randomization procedures and the completeness of outcome reporting. Each study was categorized as having low, moderate, or high risk of bias, and this classification was incorporated into the overall interpretation of findings.

Results

Study Selection

**Figure 1:**
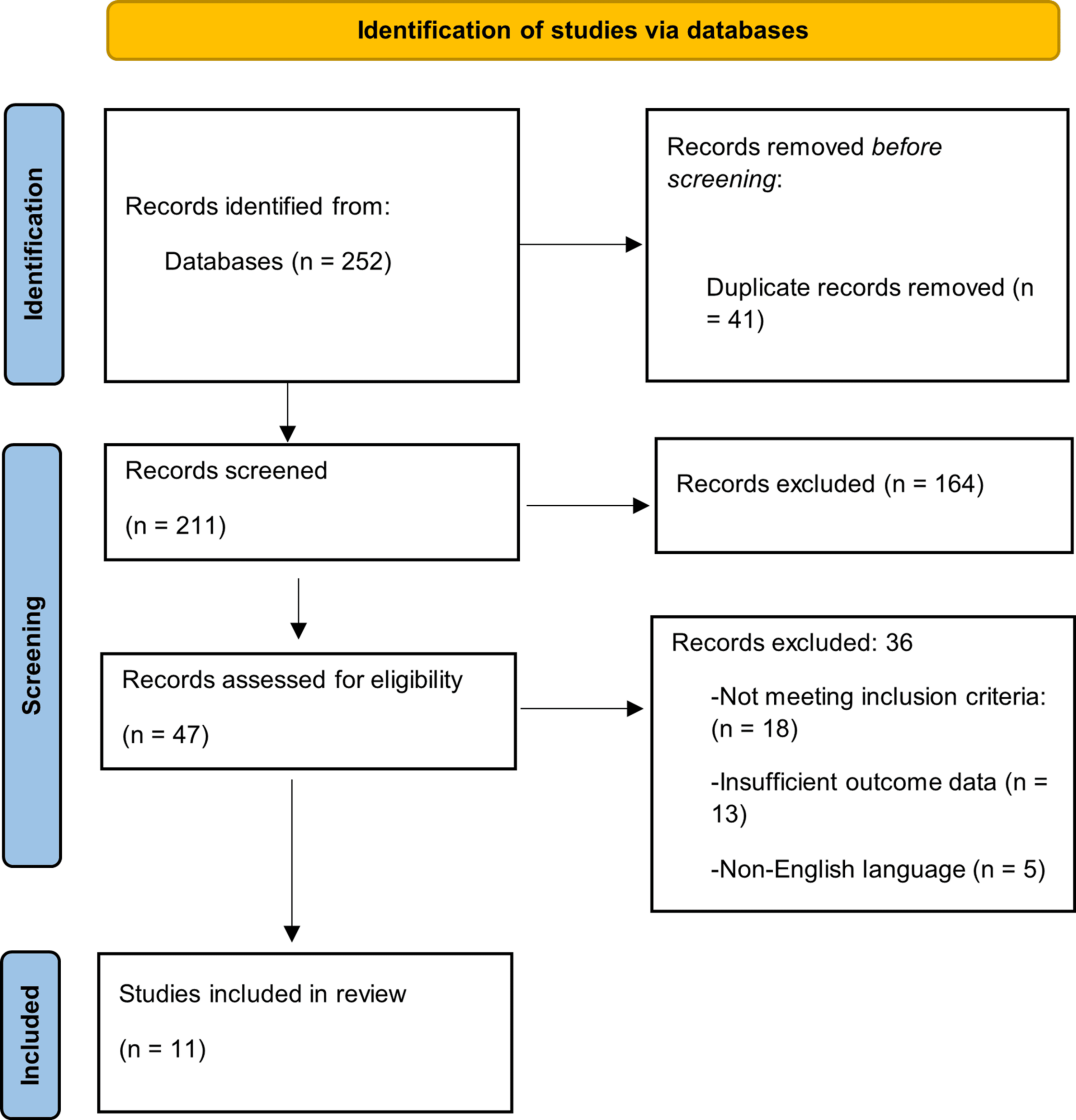
PRISMA flowchart PRISMA, Preferred Reporting Items for Systematic Reviews and Meta-Analyses

A total of 252 records were identified through the initial database search. After removal of 41 duplicate records, 211 unique records were screened by title and abstract. Of these, 164 records were excluded because they were not relevant to the review question. The remaining 47 full-text articles were assessed for eligibility. Following full-text review, 36 articles were excluded: 18 did not meet the inclusion criteria, 13 had insufficient cardiovascular outcome data, and 5 were non-English publications. These exclusion categories were applied hierarchically, and each excluded article was assigned one primary reason for exclusion to avoid overlap between categories. This selection process resulted in a final sample of 11 studies included in the review. Although the final number of included studies was relatively small, this reflected the use of predefined eligibility criteria requiring adult diabetes populations, clear cardiovascular endpoints, and extractable clinical outcome data rather than an overly restrictive search strategy. The search covered four major databases, used broad diabetes and cardiovascular search terms, and included manual reference-list screening to improve retrieval of relevant studies. The study selection process was conducted in accordance with PRISMA 2020 guidelines and is illustrated in the PRISMA flow diagram (Figure 1).

Study Characteristics

The published studies included were mainly randomized controlled trials and large-sample, multicenter cohort-based analyses. Significant evidence was provided by major trials such as GRADE, EMPA-REG OUTCOME, CANVAS, FIGARO-DKD, EXSCEL, and DCCT/EDIC. The sample sizes ranged between a few hundred and a total of over 13,000 participants, and follow-up was up to a few years. The main study populations were those who had type 2 diabetes, though there were some who had type 1 diabetes. The cardiovascular outcomes were MACE, heart failure hospitalization, cardiovascular mortality, and composite cardiorenal outcomes. Table 1 shows the key characteristics and main cardiovascular findings of the included studies.

**Table 1:** Key comparative findings of included studies on CV outcomes in diabetes mellitus ASCVD, atherosclerotic cardiovascular disease; BP, blood pressure; CABG, coronary artery bypass grafting; CKD, chronic kidney disease; CV, cardiovascular; CVD, cardiovascular disease; EF, ejection fraction; GLP-1, glucagon-like peptide-1; HF, heart failure; HFrEF, heart failure with reduced ejection fraction; MI, myocardial infarction; MRI, magnetic resonance imaging; RCT, randomized controlled trial; RV, right ventricle; SGLT2, sodium-glucose cotransporter 2; T1DM, type 1 diabetes mellitus; T2DM, type 2 diabetes mellitus

Study	Intervention/Exposure	Study Design and Population	Follow-up	Main Outcomes Assessed	Outcome Type	Standardized Effect Size	Key Findings
Marso et al. [15]	Liraglutide, GLP-1 receptor agonist	Multicenter, double-blind RCT; 9,340 patients with T2DM at high CV risk	Median 3.8 years	Composite of CV death, nonfatal MI, or nonfatal stroke	Primary	3-point MACE: HR 0.87, 95% CI 0.78–0.97. CV death: HR 0.78, 95% CI 0.66–0.93	Liraglutide significantly reduced major cardiovascular events and CV mortality compared with placebo.
Agarwal et al. [16]	Finerenone, nonsteroidal mineralocorticoid receptor antagonist	Prespecified pooled individual-patient analysis of FIDELIO-DKD and FIGARO-DKD phase III RCTs; 13,026 patients with T2DM and CKD	Median 3.0 years	Composite CV outcome; composite kidney outcome	Primary and key secondary pooled outcomes	Composite CV outcome: HR 0.86, 95% CI 0.78–0.95. Composite kidney outcome: HR 0.77, 95% CI 0.67–0.88	Finerenone significantly reduced clinically important CV and kidney outcomes across the CKD spectrum in T2DM.
Voors et al. [17]	Empagliflozin, SGLT2 inhibitor	Multinational, double-blind RCT; 530 patients hospitalized for acute de novo or decompensated HF, with or without diabetes	90 days	Hierarchical clinical benefit: death, HF events, time to first HF event, and KCCQ symptom change	Primary	Win ratio 1.36, 95% CI 1.09–1.68	Empagliflozin improved post-hospitalization clinical benefit regardless of EF or diabetes status.
Rådholm et al. [18]	Canagliflozin, SGLT2 inhibitor	CANVAS Program RCT analysis; 10,142 patients with T2DM and high CV risk	Mean 188 weeks	CV death or hospitalized HF; hospitalized HF	Primary for HF analysis; secondary CV outcomes	CV death or hospitalized HF: HR 0.78, 95% CI 0.67–0.91. Hospitalized HF: HR 0.67, 95% CI 0.52–0.87	Canagliflozin reduced CV death or HF hospitalization across patient subgroups, with greater apparent benefit among patients with baseline HF.
Zhang et al. [19]	Diabetes status and ventricular function	Retrospective observational MRI feature-tracking study; 249 HFrEF patients, including hypertensive HFrEF with and without diabetes	Cross-sectional imaging analysis; no longitudinal event follow-up reported	RV strain, LV strain, biventricular remodeling	Imaging outcomes; observational	DM associated with impaired RVGCS: β = 0.22, P = 0.004. RVGLS: β = 0.29, P < 0.001. Adjusted model RVGCS: β = 0.14, P = 0.048. RVGLS: β = 0.14, P = 0.035	Diabetes was independently associated with worse RV strain and adverse biventricular remodeling in hypertensive HFrEF.
Verma et al. [20]	Empagliflozin after CABG, SGLT2 inhibitor	Post hoc subanalysis of EMPA-REG OUTCOME RCT; T2DM with established CVD and self-reported prior CABG	Median 3.1 years	CV death, all-cause mortality, HF hospitalization, incident/worsening nephropathy	Post hoc subgroup outcomes	CV death: HR 0.52, 95% CI 0.32–0.84. All-cause mortality: HR 0.57, 95% CI 0.39–0.83. HF hospitalization: HR 0.50, 95% CI 0.32–0.77. Nephropathy: HR 0.65, 95% CI 0.50–0.84	Empagliflozin was associated with lower CV mortality, all-cause mortality, HF hospitalization, and renal events in T2DM patients with prior CABG.
Jeong et al. [21]	DASH diet and fruit/vegetable diet	Secondary analysis of randomized DASH feeding trial; 459 adults without CVD or diabetes medication use; 437 with complete lipid/BP data	8 weeks	10-year ASCVD risk estimated by pooled cohort equation; BP and lipids	Primary outcome of secondary analysis	DASH vs control: relative ASCVD risk change −10.3%, 95% CI −14.4 to −5.9. F/V vs control: −9.9%, 95% CI −14.0 to −5.5	DASH and F/V dietary patterns reduced estimated 10-year ASCVD risk by approximately 10%, largely through SBP reduction.
Neves et al. [22]	Once-weekly exenatide, GLP-1 receptor agonist	Post hoc EXSCEL trial analysis; T2DM patients with available baseline LVEF data, n = 4,749	Median 3.2 years in EXSCEL	HF hospitalization by LVEF; MACE, CV death, all-cause mortality	Main post hoc outcome: HF hospitalization; secondary CV outcomes	HHF in LVEF <40%: HR 1.52, 95% CI 0.95–2.43. HHF in LVEF ≥40%: HR 0.74, 95% CI 0.55–1.01; interaction P = 0.012	Exenatide’s HF effect differed by LVEF, suggesting possible reduced HHF risk when LVEF ≥40% and possible increased HHF risk when LVEF <40%; LVEF did not modify MACE effects.
Filippatos et al. [23]	Finerenone, FIGARO-DKD	Prespecified FIGARO-DKD HF analysis; 7,352 patients with T2DM and albuminuric CKD, excluding symptomatic HFrEF	Median 3.4 years	New-onset HF, first HHF, CV death or HHF, total HHF	Prespecified HF outcomes	New-onset HF: HR 0.68, 95% CI 0.50–0.93. First HHF: HR 0.71, 95% CI 0.56–0.90. CV death or first HHF: HR 0.82, 95% CI 0.70–0.95. Total HHF: rate ratio 0.70, 95% CI 0.52–0.94	Finerenone reduced incident HF and HF-related outcomes in T2DM with CKD, independent of baseline HF history.
GRADE Study Group [24]	Glargine, glimepiride, liraglutide, or sitagliptin added to metformin	Comparative-effectiveness RCT; 5,047 patients with T2DM	Mean 5.0 years	Microvascular outcomes, MACE, HF hospitalization, any CVD, death	Prespecified secondary outcomes	Any CVD vs other treatments: glargine HR 1.1, 95% CI 0.9–1.3; glimepiride HR 1.1, 95% CI 0.9–1.4; liraglutide HR 0.7, 95% CI 0.6–0.9; sitagliptin HR 1.2, 95% CI 1.0–1.5	Microvascular outcomes and mortality did not materially differ across treatments; liraglutide showed lower “any CVD” incidence compared with pooled alternatives.
Sousa et al. [25]	Glycemic control and cardiac autoimmunity	DCCT/EDIC cohort-based study; T1DM patients stratified by mean HbA1c ≥9.0% versus ≤7.0%	Median 26 years for CVD events	Cardiac autoantibodies, CAC, hsCRP, CVD events	Observational biomarker and clinical outcomes	≥2 Cardiac autoantibodies and CVD events: HR 16.1, 95% CI 3.0–88.2. ≥2 autoantibodies and detectable CAC: adjusted OR 60.1, 95% CI 8.8–410.0	Poor glycemic control in T1DM was linked to cardiac autoimmunity; multiple cardiac autoantibodies predicted later CAC, inflammation, and CVD events.

Glycemic Control and Cardiovascular Outcomes

Evidence from longitudinal and randomized studies suggests that glycemic control is an important but not sufficient determinant of cardiovascular risk in diabetes. In type 1 diabetes, poor glycemic control, particularly HbA1c levels above 8.0%, was associated with increased inflammatory activity, coronary artery calcification, and long-term cardiovascular risk, whereas commonly recommended glycemic targets are generally around HbA1c <7.0% in many adult patients when safely achievable [25]. However, the GRADE trial showed that different glucose-lowering strategies did not produce major differences in cardiovascular outcomes despite differences in glycemic control [24]. This finding indicates that cardiovascular risk reduction in diabetes cannot be explained by HbA1c lowering alone and is likely influenced by additional mechanisms, including inflammation, endothelial dysfunction, atherosclerosis, renal impairment, heart failure risk, and baseline cardiovascular comorbidity. Therefore, glycemic control should be interpreted as one component of cardiovascular risk management rather than as the sole therapeutic target.

Mechanistic Insights and Disease Progression

Some studies highlighted specific mechanisms linking diabetes mellitus with cardiovascular disease. Chronic hyperglycemia may promote oxidative stress through increased reactive oxygen species generation, endothelial nitric oxide depletion, and vascular endothelial injury, contributing to coronary calcification and long-term cardiovascular risk. Myocardial injury and cardiac autoimmunity were also associated with inflammatory activation in diabetes. Imaging-based evidence further showed that diabetes is associated with right ventricular dysfunction, impaired ventricular strain, and adverse cardiac remodeling, indicating that metabolic abnormalities contribute to progressive myocardial dysfunction. These findings suggest that cardiovascular complications in diabetes arise through interacting metabolic, inflammatory, vascular, and myocardial remodeling pathways. Table 2 summarizes these mechanisms and their therapeutic implications.

**Table 2:** Mechanisms and therapeutic implications in cardiovascular disease in diabetes ASCVD, atherosclerotic cardiovascular disease; GLP-1, glucagon-like peptide-1; SGLT2, sodium-glucose cotransporter 2

Domain	Description	Study Reference
Metabolic mechanisms	Chronic hyperglycemia promotes oxidative stress through increased reactive oxygen species generation, endothelial nitric oxide depletion, and vascular injury	[25]
Cardiac structural changes	Diabetes is associated with impaired ventricular strain, right ventricular dysfunction, and adverse cardiac remodeling	[19]
Inflammatory pathways	Inflammatory activation and cardiac autoimmunity may contribute to coronary calcification and long-term cardiovascular risk	[25]
Pharmacological intervention (SGLT2 inhibitors)	SGLT2 inhibitors reduce heart failure events and renal progression, likely through hemodynamic, natriuretic, and metabolic effects beyond glucose lowering	[17,20]
Pharmacological intervention (GLP-1 agonists)	GLP-1 receptor agonists reduce major cardiovascular events, potentially through weight reduction, blood pressure effects, anti-inflammatory activity, and anti-atherosclerotic mechanisms	[15]
Mineralocorticoid receptor antagonism	Finerenone reduces cardiorenal outcomes by limiting mineralocorticoid receptor-mediated inflammation and fibrosis	[16,23]
Lifestyle modification	Dietary interventions improve cardiovascular risk profiles through reductions in blood pressure, lipid parameters, and estimated ASCVD risk	[21]
Treatment heterogeneity	Cardiovascular benefit varies according to baseline cardiac function, including ejection fraction and heart failure status	[22]
Residual cardiovascular risk	Persistent cardiovascular risk remains despite standard therapies, suggesting incomplete control of inflammatory, vascular, renal, and myocardial pathways	[18]

Cardiovascular Outcomes and Complications

Diabetes mellitus was constantly accompanied by a higher risk of cardiovascular complications, such as coronary artery disease, stroke, and heart failure. Studies carried out by imaging proved that diabetic patients had poor ventricular function with lower global strain parameters and both left and right ventricular dysfunction. Diabetes was an independent variable that was associated with poor right ventricular function and poor ventricular interdependence in hypertensive patients with heart failure. These results show that diabetes worsens cardiac dysfunction and leads to the development of disease in patients with pre-existing cardiovascular conditions. Table 3 shows the cardiovascular outcomes that were reported in the included studies.

**Table 3:** CV outcomes reported across included studies ASCVD, atherosclerotic cardiovascular disease; CABG, coronary artery bypass grafting; CV, cardiovascular; EF, ejection fraction; GLP-1, glucagon-like peptide-1; HF, heart failure; MACE, major adverse cardiovascular events; MI, myocardial infarction

Outcome Category	Key Findings	Study Reference
MACE	Reduction in composite outcomes, including CV death, MI, and stroke, with GLP-1 receptor agonists	[15]
Cardiovascular and renal composite outcomes	Significant reduction in CV events and kidney disease progression with finerenone	[16]
Acute heart failure outcomes	Improved survival and reduced HF events with empagliflozin in hospitalized patients	[17]
Heart failure hospitalization	Reduced HF hospitalization and CV death with canagliflozin	[18]
Ventricular dysfunction	Diabetes is associated with impaired right ventricular function and cardiac remodeling.	[19]
Post-surgical cardiovascular outcomes	Reduced CV mortality and HF events with empagliflozin post-CABG	[20]
Long-term ASCVD risk	Dietary interventions reduced the estimated 10-year cardiovascular risk	[21]
Glycemic control and CV outcomes	No significant difference in CV outcomes across glycemic strategies	[24]
Inflammatory and autoimmune mechanisms	Poor glycemic control linked to increased CV risk via inflammation	[25]
Incident heart failure risk	Reduced risk of new-onset HF with finerenone therapy	[23]
Ejection fraction-based outcomes	Variable CV benefit of GLP-1 therapy based on baseline EF	[22]

*Pharmacological Interventions and Cardiovascular Benefit*s

Multiple randomized trials evaluated the cardiovascular effects of antidiabetic therapies. Sodium-glucose cotransporter 2 (SGLT2) inhibitors reduced cardiovascular mortality, heart failure hospitalization, and renal disease progression. The main EMPA-REG OUTCOME trial is cited as background evidence, whereas the included EMPA-REG OUTCOME evidence in this review was the post-CABG (coronary artery bypass grafting) subanalysis by Verma et al. [20]. In EMPA-REG OUTCOME, empagliflozin reduced cardiovascular death by 38% (HR 0.62), all-cause mortality by 32% (HR 0.68), and heart failure hospitalization by 35% (HR 0.65). Similarly, canagliflozin reduced cardiovascular death or hospitalized heart failure by 22% in CANVAS (HR 0.78; 95% CI 0.67-0.91).

Glucagon-like peptide-1 (GLP-1) receptor agonists also demonstrated cardiovascular benefits. Liraglutide reduced MACE by 13% in LEADER (HR 0.87; 95% CI 0.78-0.97), whereas EXSCEL showed that exenatide outcomes varied by baseline ejection fraction. Finerenone reduced composite cardiovascular outcomes by 14% in FIDELITY (HR 0.86; 95% CI 0.78-0.95) and incident heart failure by 29% in FIGARO-DKD (HR 0.71; 95% CI 0.56-0.90). Overall, SGLT2 inhibitors showed stronger heart failure-related benefits, GLP-1 receptor agonists mainly reduced atherosclerotic events, and finerenone provided cardiorenal protection in diabetic kidney disease.

Heart Failure Outcomes in Diabetes

Heart failure emerged as a major complication in diabetic populations across multiple studies. Clinical trials demonstrated that newer pharmacological agents significantly reduced the incidence of heart failure events and hospitalizations. In patients hospitalized for acute heart failure, initiation of empagliflozin resulted in improved clinical outcomes, including reduced mortality and heart failure events within a short follow-up period. Additionally, studies involving patients with chronic kidney disease and diabetes showed that targeted therapies reduced the risk of new-onset heart failure and improved overall cardiovascular outcomes.

Lifestyle and Risk Modification

Lifestyle interventions also contributed to cardiovascular risk reduction. Evidence from dietary intervention trials demonstrated that structured dietary patterns, such as the DASH diet, significantly reduced estimated 10-year cardiovascular risk through improvements in blood pressure and lipid profiles. These findings highlight the role of non-pharmacological strategies in mitigating cardiovascular risk in populations at risk, including those with metabolic disorders.

Comparative Analysis Across Studies

**Figure 2:**
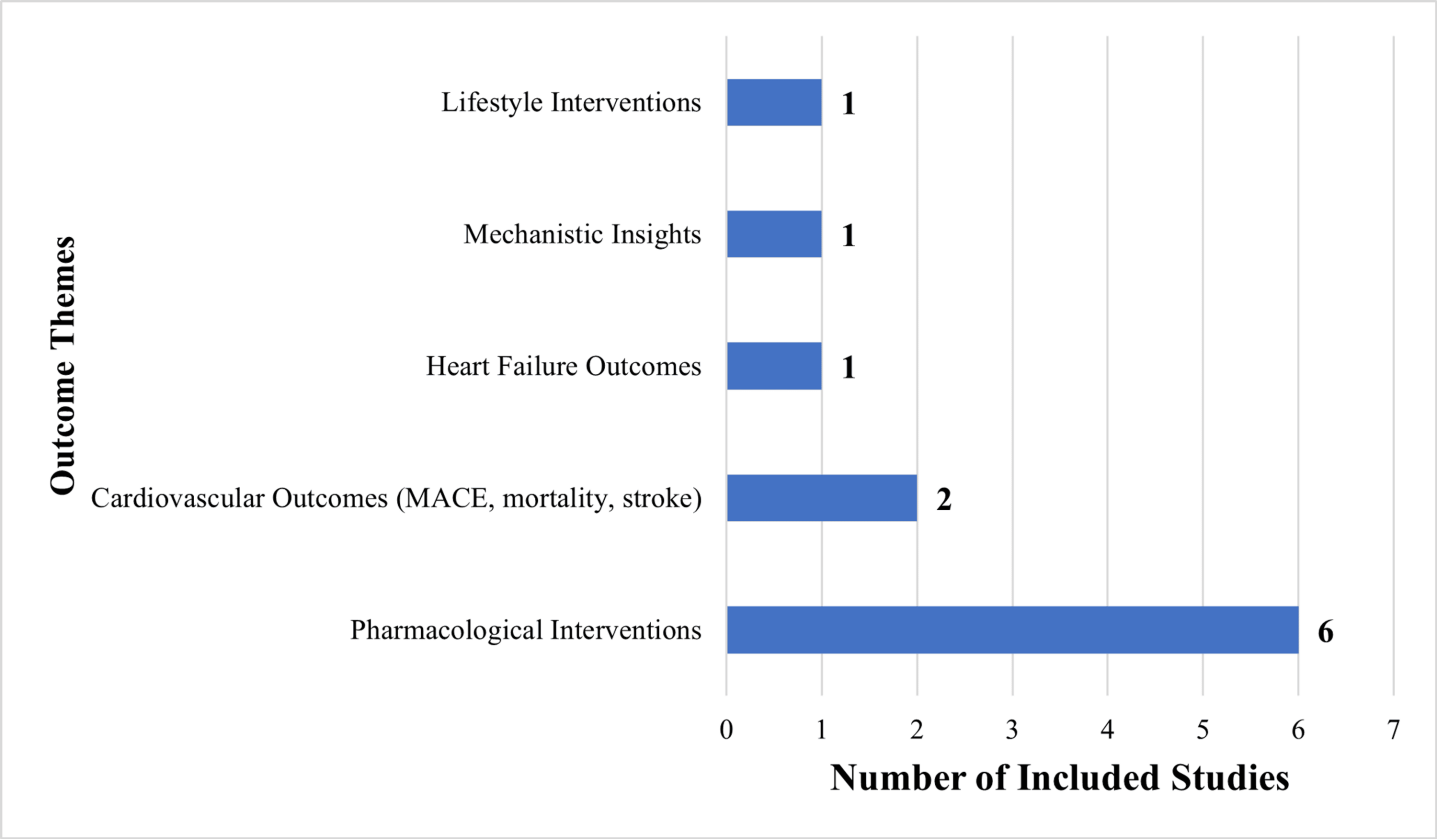
Distribution of included studies by outcome themes MACE, major adverse cardiovascular events

Across the included studies, consistent patterns were observed regarding the increased cardiovascular risk associated with diabetes and the benefits of specific therapeutic interventions. While pharmacological treatments such as SGLT2 inhibitors and GLP-1 receptor agonists showed favorable cardiovascular outcomes, variability was noted depending on baseline patient characteristics, comorbid conditions, and study design. Some glucose-lowering therapies demonstrated neutral effects on cardiovascular outcomes, indicating heterogeneity in treatment responses. Figure 2 shows the distribution of included studies across different outcome themes.

Quality Assessment Findings

The methodological quality of the included studies was variable but stronger among randomized controlled trials than observational or subgroup analyses. Based on the risk-of-bias assessment, seven studies were low risk, one was low-to-moderate risk, and three were moderate risk; no studies were high risk. Moderate-risk ratings mainly reflected observational designs, subgroup/post hoc analyses, potential confounding, and variation in outcome definitions.

Risk-of-Bias Assessment

The risk of bias between studies was mainly low to medium. Internal validity was good in the randomized controlled trials that had the right randomization and outcome reporting. Nonetheless, other studies included subgroup or post hoc analysis, which brought about the possibility of bias. Observational elements were more prone to confounding effects, though attempts were made to control the differences at the baseline. Table 4 shows the risk of bias of the included studies.

**Table 4:** Risk-of-bias assessment of included studies RCT, randomized controlled trial

Study	Study Design	Selection Bias	Performance Bias	Detection Bias	Overall Risk of Bias
Marso et al. [15]	RCT	Low	Low	Low	Low
Agarwal et al. [16]	Pooled RCT analysis	Low	Low	Low	Low
Voors et al. [17]	RCT	Low	Low	Low	Low
Rådholm et al. [18]	RCT	Low	Low	Low	Low
Zhang et al. [19]	Observational study	Moderate	Moderate	Moderate	Moderate
Verma et al. [20]	RCT subanalysis	Low	Low	Moderate	Low-moderate
Jeong et al. [21]	Randomized trial	Low	Low	Low	Low
GRADE Study Group [24]	RCT	Low	Low	Low	Low
Sousa et al. [25]	Cohort study	Moderate	Moderate	Moderate	Moderate
Filippatos et al. [23]	RCT	Low	Low	Low	Low
Neves et al. [22]	RCT subgroup analysis	Low	Moderate	Moderate	Moderate

Discussion

The current review suggests that diabetes mellitus is associated with increased cardiovascular burden through interacting mechanisms of metabolic dysfunction, endothelial injury, inflammation, atherosclerosis, and cardiac remodeling. Across the included studies, diabetes was linked with adverse cardiovascular outcomes, including MACE, heart failure hospitalization, cardiovascular mortality, ventricular dysfunction, and cardiorenal complications. Because the included evidence was heterogeneous and synthesized narratively, these findings should be interpreted as an integrated summary rather than a pooled quantitative estimate. Pharmacological evidence suggested that cardiovascular benefit differed by drug class and patient phenotype. SGLT2 inhibitors showed consistent benefit for heart failure-related outcomes, with empagliflozin reducing cardiovascular death by 38% and heart failure hospitalization by 35% in EMPA-REG OUTCOME and canagliflozin reducing cardiovascular death or hospitalized heart failure by 22% in CANVAS. GLP-1 receptor agonists appeared to provide greater benefit for atherosclerotic cardiovascular outcomes, with liraglutide reducing MACE by 13% in LEADER. Finerenone provided cardiorenal benefit in patients with type 2 diabetes and chronic kidney disease, reducing composite cardiovascular outcomes by 14% in FIDELITY and incident heart failure by 29% in FIGARO-DKD.

Residual cardiovascular risk appeared most relevant in patients with established cardiovascular disease, chronic kidney disease, heart failure, poor glycemic control, albuminuria, or evidence of vascular or myocardial remodeling. This persistent risk may indicate that cardiovascular complications in diabetes are not explained by hyperglycemia alone but also reflect inflammation, endothelial dysfunction, renal impairment, plaque instability, and cardiac structural changes. Treatment strategies may therefore require integration of glycemic control with therapies targeting heart failure, atherosclerotic risk, renal protection, and lifestyle-related risk factors. Studies outside the 11 included articles are discussed only as narrative background and were not treated as part of the systematic evidence synthesis. Recent literature provides contextual support for mechanisms and therapies not included as primary evidence in this review [26-28]. Metformin’s potential cardioprotective effects are discussed as background context rather than as a finding from the 11 included studies. Similarly, broader evidence on metabolic inflexibility, mitochondrial dysfunction, lipid metabolism, vascular calcification, albuminuria, and lifestyle modification provides biological context for persistent cardiovascular risk in diabetes [11,12,29].

Overall, the narrative synthesis indicates that diabetes-related cardiovascular risk is multifactorial and may not be fully addressed by glucose-lowering alone. SGLT2 inhibitors, GLP-1 receptor agonists, and finerenone appear to provide complementary cardiovascular benefits, but their effects differ across heart failure, atherosclerotic, renal, and residual-risk domains. Future research should clarify which patient subgroups derive the greatest benefit from each therapeutic class and define strategies to reduce residual cardiovascular risk despite contemporary standard therapy.

Limitations and Future Directions

There are some limitations of the current review. The included studies were heterogeneous in design, population, interventions, follow-up duration, and cardiovascular outcome definitions, which prevented quantitative synthesis. A meta-analysis was not performed; therefore, the findings should be interpreted as a narrative synthesis rather than pooled quantitative evidence. The final evidence base was limited, with 11 included studies, and restriction to four databases and English-language publications may affect generalizability and introduce publication or language bias. Potential selection bias may also be present because of the eligibility criteria and database selection. No review protocol was prospectively registered. The inclusion of randomized trials, observational studies, subgroup analyses, and post hoc analyses also introduced methodological variability. Findings from subgroup and post hoc analyses were interpreted cautiously because these analyses are generally less definitive than primary trial outcomes.

Future research should include larger, standardized randomized trials with longer follow-up and consistent cardiovascular endpoints. Further comparative studies across therapeutic classes and patient subgroups may help clarify treatment response in patients with heart failure, chronic kidney disease, albuminuria, established cardiovascular disease, and persistent residual cardiovascular risk. Incorporation of biomarkers, imaging, and real-world data may improve early risk stratification and support more individualized cardiovascular prevention strategies in diabetes.

## Conclusions

Diabetes mellitus is a major contributor to cardiovascular morbidity and mortality through complex interactions among metabolic dysfunction, vascular injury, inflammation, atherosclerosis, and cardiac remodeling. Based on the included studies, diabetes was associated with an increased risk of MACE, heart failure hospitalization, cardiovascular mortality, ventricular dysfunction, and cardiorenal complications. Mechanistic evidence suggests that hyperglycemia, endothelial dysfunction, oxidative stress, and chronic inflammation contribute to atherosclerosis and myocardial remodeling. Therapeutic findings from the included studies indicate that SGLT2 inhibitors were most consistently associated with reductions in heart failure hospitalization and cardiovascular mortality, GLP-1 receptor agonists were associated with reductions in MACE, and finerenone showed cardiorenal benefit in patients with type 2 diabetes and chronic kidney disease. Lifestyle interventions, including dietary modification, may also improve cardiovascular risk profiles. Personalized management should therefore consider patient phenotype, such as heart failure status, ejection fraction, chronic kidney disease, albuminuria, baseline cardiovascular risk, and treatment response, when selecting therapies such as SGLT2 inhibitors, GLP-1 receptor agonists, or mineralocorticoid receptor antagonists. Future research should prioritize comparative studies that define which patient subgroups derive the greatest cardiovascular benefit from each therapeutic class and how residual cardiovascular risk can be reduced despite contemporary standard care.

## References

[1] Islam K, Islam R, Nguyen I (2025). Diabetes mellitus and associated vascular disease: pathogenesis, complications, and evolving treatments. Adv Ther.

[2] Wong ND, Sattar N (2023). Cardiovascular risk in diabetes mellitus: epidemiology, assessment and prevention. Nat Rev Cardiol.

[3] Leong DP, Joseph PG, McKee M, Anand SS, Teo KK, Schwalm JD, Yusuf S (2017). Reducing the global burden of cardiovascular disease, part 2: prevention and treatment of cardiovascular disease. Circ Res.

[4] Luo F, Das A, Chen J, Wu P, Li X, Fang Z (2019). Metformin in patients with and without diabetes: a paradigm shift in cardiovascular disease management. Cardiovasc Diabetol.

[5] Diniz MS, Hiden U, Falcão-Pires I, Oliveira PJ, Sobrevia L, Pereira SP (2023). Fetoplacental endothelial dysfunction in gestational diabetes mellitus and maternal obesity: a potential threat for programming cardiovascular disease. Biochim Biophys Acta Mol Basis Dis.

[6] Stehouwer CD, Nauta JJ, Zeldenrust GC, Hackeng WH, Donker AJ, den Ottolander GJ (1992). Urinary albumin excretion, cardiovascular disease, and endothelial dysfunction in non-insulin-dependent diabetes mellitus. Lancet.

[7] Leon BM, Maddox TM (2015). Diabetes and cardiovascular disease: Epidemiology, biological mechanisms, treatment recommendations and future research. World J Diabetes.

[8] Wang M, Li Y, Li S, Lv J (2022). Endothelial dysfunction and diabetic cardiomyopathy. Front Endocrinol (Lausanne).

[9] Su J, Luo Y, Hu S, Tang L, Ouyang S (2023). Advances in research on type 2 diabetes mellitus targets and therapeutic agents. Int J Mol Sci.

[10] Erdélyi A, Pálfi E, Tűű L (2023). The importance of nutrition in menopause and perimenopause-a review. Nutrients.

[11] Poznyak A, Grechko AV, Poggio P, Myasoedova VA, Alfieri V, Orekhov AN (2020). The Diabetes mellitus-atherosclerosis connection: the role of lipid and glucose metabolism and chronic inflammation. Int J Mol Sci.

[12] Page MJ, McKenzie JE, Bossuyt PM (2021). The PRISMA 2020 statement: an updated guideline for reporting systematic reviews. BMJ.

[13] Sterne JA, Savović J, Page MJ (2019). RoB 2: a revised tool for assessing risk of bias in randomised trials. BMJ.

[14] Gualdi-Russo E, Zaccagni L (2026). The Newcastle-Ottawa Scale for assessing the quality of studies in systematic reviews. Publications.

[15] Marso SP, Daniels GH, Brown-Frandsen K (2016). Liraglutide and cardiovascular outcomes in type 2 diabetes. N Engl J Med.

[16] Agarwal R, Filippatos G, Pitt B (2022). Cardiovascular and kidney outcomes with finerenone in patients with type 2 diabetes and chronic kidney disease: the FIDELITY pooled analysis. Eur Heart J.

[17] Voors AA, Angermann CE, Teerlink JR (2022). The SGLT2 inhibitor empagliflozin in patients hospitalized for acute heart failure: a multinational randomized trial. Nat Med.

[18] Rådholm K, Figtree G, Perkovic V (2018). Canagliflozin and heart failure in type 2 diabetes mellitus: results from the CANVAS program. Circulation.

[19] Zhang G, Shi R, Li XM (2024). Impact of diabetes mellitus on right ventricular dysfunction and ventricular interdependence in hypertensive patients with heart failure with reduced ejection fraction assessed via 3.0 T cardiac MRI. Cardiovasc Diabetol.

[20] Verma S, Mazer CD, Fitchett D, Inzucchi SE, Pfarr E, George JT, Zinman B (2018). Empagliflozin reduces cardiovascular events, mortality and renal events in participants with type 2 diabetes after coronary artery bypass graft surgery: subanalysis of the EMPA-REG OUTCOME® randomised trial. Diabetologia.

[21] Jeong SY, Wee CC, Kovell LC (2023). Effects of diet on 10-year atherosclerotic cardiovascular disease risk (from the DASH trial). Am J Cardiol.

[22] Neves JS, Leite AR, Mentz RJ (2025). Cardiovascular outcomes with exenatide in type 2 diabetes according to ejection fraction: The EXSCEL trial. Eur J Heart Fail.

[23] Filippatos G, Anker SD, Agarwal R (2022). Finerenone reduces risk of incident heart failure in patients with chronic kidney disease and type 2 diabetes: analyses from the FIGARO-DKD trial. Circulation.

[24] Nathan DM, Lachin JM, Bebu I (2022). Glycemia reduction in type 2 diabetes - microvascular and cardiovascular outcomes. N Engl J Med.

[25] Sousa GR, Pober D, Galderisi A (2019). Glycemic control, cardiac autoimmunity, and long-term risk of cardiovascular disease in type 1 diabetes mellitus. Circulation.

[26] Młynarska E, Czarnik W, Dzieża N (2025). Type 2 diabetes mellitus: new pathogenetic mechanisms, treatment and the most important complications. Int J Mol Sci.

[27] Geum MJ, Oh KS, Ah YM (2026). Sodium-glucose cotransporter 2 inhibitors in diabetic solid organ transplant recipients: a systematic review and meta-analysis of comparative studies. J Diabetes Res.

[28] Laakso M, Fernandes Silva L (2023). Statins and risk of type 2 diabetes: mechanism and clinical implications. Front Endocrinol (Lausanne).

[29] Kawai Y, Uneda K, Yamada T (2022). Comparison of effects of SGLT-2 inhibitors and GLP-1 receptor agonists on cardiovascular and renal outcomes in type 2 diabetes mellitus patients with/without albuminuria: a systematic review and network meta-analysis. Diabetes Res Clin Pract.

